# Recent Advances in Dopamine Receptor Ligands as Chemical Biology Tools

**DOI:** 10.1021/acsomega.6c01474

**Published:** 2026-06-10

**Authors:** Michael Dorogan, Hari K. Namballa, Sandip Patra, Wayne W. Harding

**Affiliations:** † Department of Chemistry, 6614 University of Pittsburgh, Chevron Science Center, 219 Parkman Avenue, Pittsburgh, Pennsylvania 15260, United States; ⊥ Department of Chemistry, Hunter College, City University of New York, 695 Park Avenue, New York, New York 10065, United States; ‡ PhD. Program in Chemistry, CUNY Graduate Center, 365 5th Avenue, New York, New York 10016, United States; § PhD. Program in Biochemistry, CUNY Graduate Center, 365 5th Avenue, New York, New York 10016, United States

## Abstract

Dopamine receptors (DRs) have been implicated in numerous disorders and diseases (e.g., Alzheimer’s disease, Parkinson’s disease, schizophrenia, and substance use disorders) and have served as attractive drug targets for these ailments. Despite their potential clinical utility, the development of selective DR ligands has been challenging due to difficulties in selectivity among the DR subtypes as well as other biogenic amine receptors and poor pharmacokinetic properties. The realization of their full potential necessitates continued advancements in DR ligands as investigative tools. This review aims to highlight the recent developments made in the chemical biology of DR ligands (e.g., bivalent ligands, photoactivatable ligands, photoswitchable probes, and fluorescent probes).

## Graphical abstract

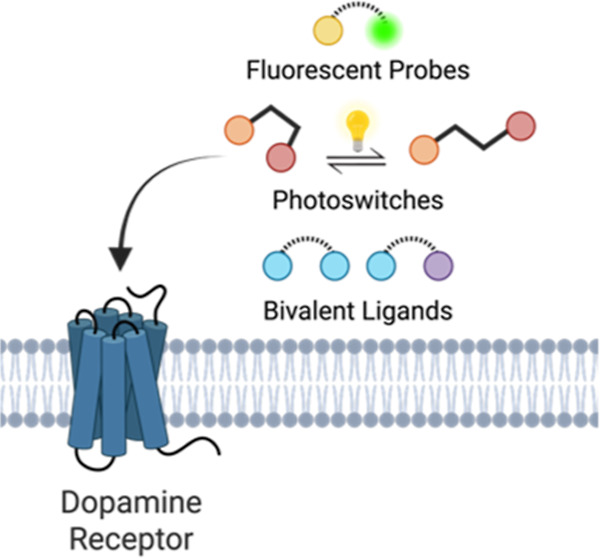

## 1 Introduction

G protein-coupled receptors (GPCRs) are a superfamily of evolutionarily related cell surface proteins, characteristically defined by their seven transmembrane domains, and display a myriad of physiological functions (ranging from vision and taste sensing to mood and behavioral regulation).^1^ All GPCRs consist of a single polypeptide chain with seven α-helical segments connected by intracellular and extracellular loops. This classical receptor unit allows for transduction and amplification of extracellular signals through the production of secondary messengers via a conformational change and interaction with intracellular G proteins upon binding an extracellular ligand (such as amines, ions, lipids, nucleotides, peptides, proteins, and photons).^2–4^ As GPCRs are implicated in numerous diseases (e.g., cancers, mental disorders, metabolic disorders, cardiovascular diseases, inflammatory diseases, and sensory disorders), it is unsurprising that over 30% of FDA-approved drugs target this superfamily of receptors.^5^

Nearly all FDA-approved drugs that act on GPCRs bind at the orthosteric site (the same binding site as the endogenous ligand) and regulate receptor function through three distinct mechanisms: classical agonism (through direct stimulation), inverse agonism (through inhibition of the constitutive activity of the receptor), or competitive antagonism (through blockage of the binding of the endogenous agonist).^6–10^ Efforts to produce highly selective ligands often fail due to the highly conserved nature of the orthosteric binding site across a family of GPCRs. Furthermore, the clinical utility of many synthetic orthosteric ligands is impeded by detrimental physicochemical and drug metabolism/pharmacokinetic (DMPK) properties. In fact, many direct-acting (orthosteric) agonists are toxic or have the potential to lead to desensitization, down-regulation, or internalization due to prolonged periods of activation.^11,12^

Alternatively, allosteric modulators bind to the topographically distinct allosteric site (anywhere other than the orthosteric active site) to either inhibit or potentiate the binding and/or signaling of an orthosteric ligand.^13^ As allosteric sites could be less preserved across receptor subtype families, allosteric ligands may have a higher degree of selectivity compared to orthosteric ligands.^14^ Allosteric modulation has gained substantial interest in modern drug discovery with the clinical success of the first allosteric modulator drugs, namely, benzodiazepines. These drugs potentiate [as positive allosteric modulators] or inhibit [as negative allosteric modulators (NAMs)] the effect of the neurotransmitter GABA at the ionotropic GABA_A_ receptor.^6,15^ These allosteric modulators have significantly better safety and tolerability profiles than orthosteric agonists (with equivalent effectiveness) in the GABAergic systems.

Functional selectivity adds additional complexity as GPCRs can undergo biased (pluripotency) signaling, as biased ligands can stabilize subsets of receptor conformations, thus enabling unique pharmacology (e.g., G protein-dependent signaling versus β-arrestin-dependent signaling) and the potential to reduce on-target adverse effects.^16,17^

As members of the GPCR superfamily, dopamine receptors (DRs) recognize the endogenous, catecholamine dopamine (DA) and are categorized into two main families: the D_1_-like family, comprising D_1_R and D_5_R, and the D_2_-like family, comprising D_2_R, D_3_R, and D_4_R.^18^ The D_1_-like family of receptors is positively coupled to adenylate cyclase (signaling through Gα_s_ or Gα_olf_ proteins), whereas the D_2_-like family of receptors is negatively coupled to adenylate cyclase (signaling through Gα _*i*/o_ proteins). Dopamine is involved in numerous behavioral and physiological processes (including movement, neuroendocrine control, executive function, motivation, and reward). DRs are recognized as therapeutic targets for the treatment of several diseases, ranging from antipsychotic disorders to Parkinson’s disease to psychostimulant addiction.

Conventional (orthosteric) DR ligands have or are predicted to have side effects when deployed in a clinical setting due to a lack of selectivity between the subtypes of DRs, as well as related biogenic amine receptors such as adrenergic or serotonergic receptors. Furthermore, some selective DR ligands are hampered by poor pharmacokinetic properties, such as poor central nervous system (CNS) penetration and limited oral bioavailability, which preclude their clinical translation. The development of DR ligands as therapeutics is further complicated by the complex multifactorial pathophysiology of neurodegenerative disorders and neurological diseases; thus, polypharmacology is an emerging therapeutic strategy.^19^

Because of the multifaceted nature of DRs, chemical tools (Figure 1) are essential to uncover new mechanistic insights and potentially lead to the development of therapeutics with novel mechanisms of action. As this Review will reveal, bivalent ligands help tease apart the complex signaling pathways of dimeric DRs, whereas photoswitchable and photoactivatable ligands enable insights into the spatiotemporal signaling of DRs. Fluorescent probes facilitate the visualization of DRs, while photoaffinity probes provide insight into binding interactions and enable proteomic analysis. The chemical tools of DRs are not designed to be therapeutic agents (a distinction from medicinal chemistry) but rather to enable a careful and controlled study of DRs. Collectively, these chemical tools play pivotal roles in elucidating the complex signaling pathways of DRs and establishing new conceptual frameworks for DR biology.

**Figure 1:**
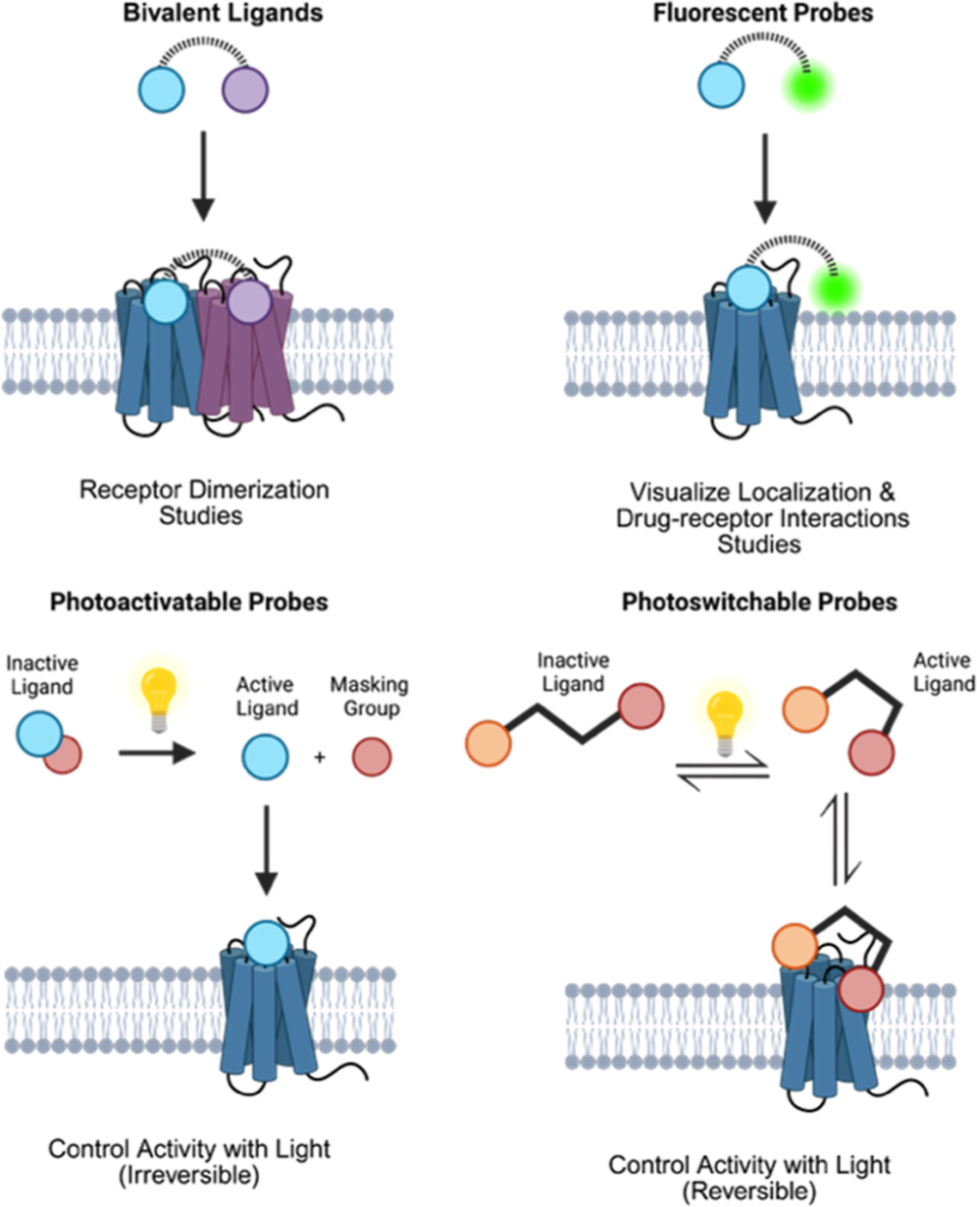
Graphical overview of key chemical biology tools.

## 2 Bivalent Ligands

An accumulating number of studies have demonstrated that GPCRs do not solely exist as isolated entities in the plasma membrane but can homodimerize and heterodimerize as well as form higher-order oligomers.^18,20–23^ Receptor oligomerization can activate alternative signaling pathways, influencing receptor trafficking, function, and ligand pharmacology.^24,25^ As DR oligomerization is implicated in disorders and diseases (e.g., schizophrenia, Parkinson’s disease, and substance abuse disorder) through dysfunctional multimers, there is growing interest in efforts to identify ligands that target specific DR heterodimer or hetero-oligomer pairs.^26–30^

Compared to a traditional (monovalent) ligand that binds to only one binding site on a target macromolecule, bivalent ligands are constructed of two pharmacophores linked by a non-cleavable spacer, enabling simultaneous binding to two targets (e.g., two protomers) (Figure 2).^31^ Bivalent ligands are classified into two classes: homobivalent ligands, with two of the same pharmacophore, and heterobivalent ligands, with two different pharmacophores. As bivalent ligands can bridge two pharmacophore moieties, they enable fundamental investigations into the GPCR dimerization process. Rather than simultaneous binding, as is the case with bivalent ligands, dual-acting ligands allow for the simultaneous delivery of both pharmacophoric moieties via either the fusion or merger (with a relatively short spacer) of two pharmacophoric units (Figure 2).^32,33^ Dual-acting ligands have potential as therapeutic agents due to improved “drug-like” properties (e.g., lower molecular mass, clogP, and topological polar surface area).^34^ Due to their large molecular size (often more than >1KDa), bivalent ligands face translational limitations such as poor permeability and pharmacokinetics; nevertheless, bivalent ligands are still useful as mechanistic probes to study GPCR oligomerization.

**Figure 2:**
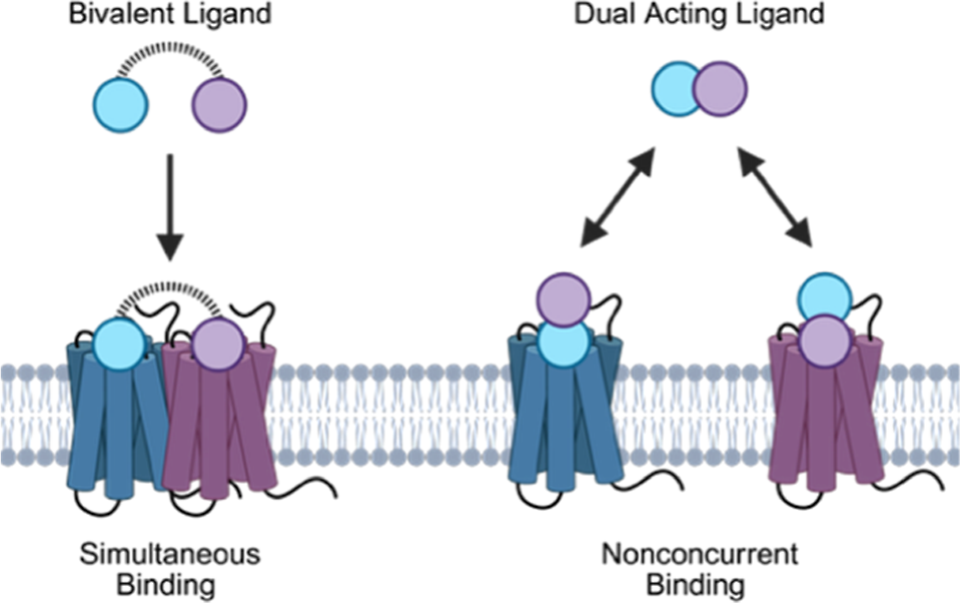
Binding mode of bivalent and dual-acting ligands.

GPCR oligomerization (and by extension, DRs oligomerization) is not without controversy, as the oligomeric status is still debated. For instance, bioluminescence resonance energy transfer (BRET) studies of β2 adrenergic receptors, which belong to the same class A (rhodopsin-like) family of GPCR as DRs, suggest they do not oligomerize in cells.^24^ Previous BRET studies relied on simplified (and often faulty) assumptions, which impaired the results. These conclusions are supported by recent single-molecule fluorescence resonance energy transfer imaging studies, which further demonstrated the possibility that these receptors do not dimerize at significant levels.^35^ Thus, bivalent ligands may simply artificially induce GPCR oligomerization or downstream (monomeric) crosstalk.

### 2.1 DR Homodimer-Targeting Ligands

Homodimers of D_2_R are implicated in the pathophysiology of schizophrenia, as the striatal tissue of animal models of schizophrenia and post-mortem striatal sections from schizophrenia patients have higher expression of D_2_ dimers, but lower expression of D_2_ monomers.^36–38^ As with other class A GPCRs, the transmembrane helices (TM) 1, 4, and 5 of D_2_R are most relevant for dimerization due to accessibility.^39,40^ Crystal structures of class A dimers reveal common orientations of head-to-head TM1/2 and TM4/5; however, crystallographic structures are generally unable to resolve N- and C-termini and long intracellular loop (ICL) 3, thus potentially missing key interactions.^41^ Through cross-linking of substituted cysteines (conserved in most rhodopsin-like GPCRs), conformational change at the TM4 dimer interface is implicated as part of the D_2_R homodimer activation mechanism. Compared to the active state, with TM4–TM4 forming the interface, the inactive inverse agonist-bound state has TM4–TM5 forming the interface.^42–44^ TM1 was identified as a second symmetrical interface of D_2_R, which is relevant for higher-order oligomerization; however, this site does not appear to undergo major conformational changes upon ligand binding.^44^

To enable an investigation into homodimers of D_2_R, a series of homodimeric dopamine D_2_R ligands were synthesized through the incorporation of the privileged structure of 1,4-disubstituted aromatic piperidines/piperazines (1,4-DAPs) and triazolyl-linked spacer elements.^45^ Bivalent ligand **1** (Figure 3) emerged as the compound with the highest Hill slope of 2.0, indicating positive cooperative binding for both D_2long_R and D_2short_R.

**Figure 3:**
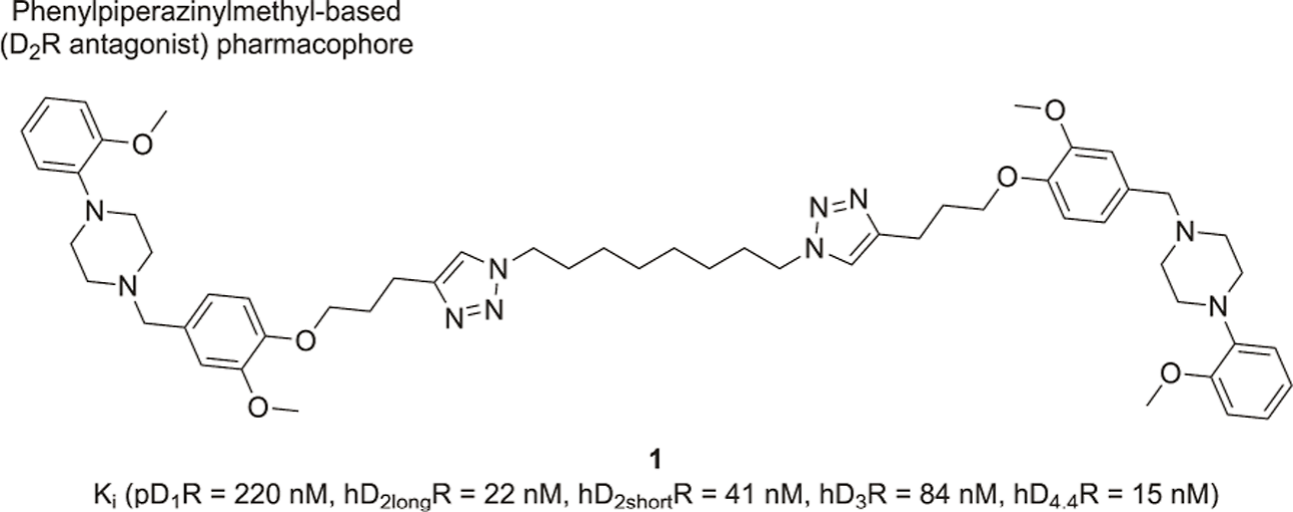
Chemical structures of phenylpiperazinylmethyl-based homobivalent ligands for D_2_R.

Similarly, bivalent D_2_R agonists were designed through the incorporation of a bis-aminoindane derivative connected by triazolyl-linked spacer units, which are useful as molecular probes to investigate pathologies such as dyskinesia, hyperprolactinemia, Parkinson’s disease, and restless legs syndrome.^46^ Both the monovalent and bivalent aminoindane derivatives **2** and **3** (Figure 4) were selective for the D_2_-like family of receptors; however, a significantly lower EC_50_ value was observed for monovalent ligand **3** (D_2long_R EC_50_ = 13 nM) compared to bivalent ligand **2** (D_2long_R EC_50_ = 440 nM). In the same study, the heterobivalent ligands (**4** and **5**), comprising aminoindane agonist and 1,4-DAP antagonist pharmacophores, revealed functional crosstalk between the two D_2_R protomers as inhibition of D_2_ internalization was observed.

**Figure 4:**
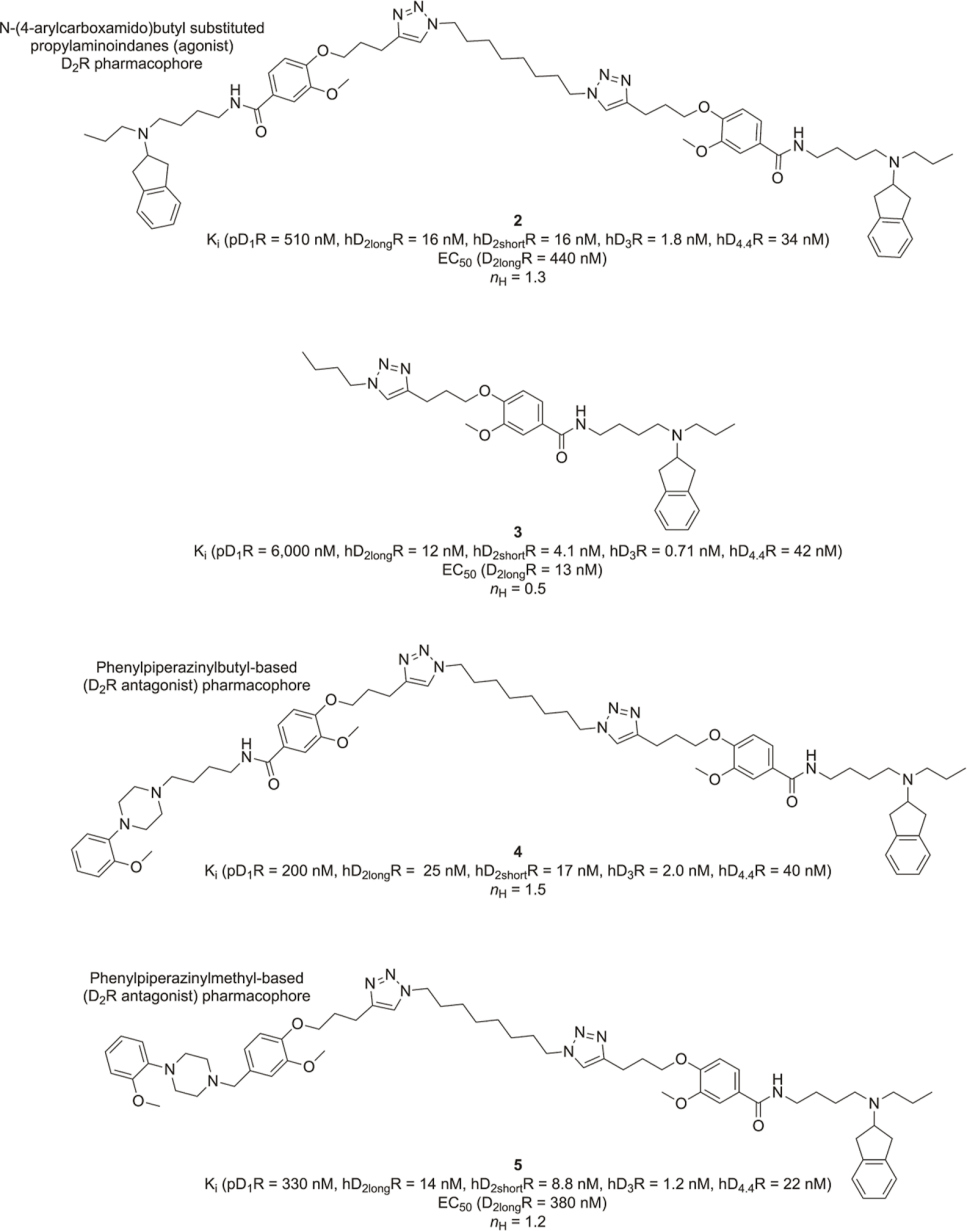
Chemical structures of *N*-(4-arylcarboxamido)­butyl-substituted propylaminoindane-based homo- and heterobivalent ligands for D_2_R and monovalent aminoindane derivative.

Homobivalent agonists targeting the D_2_R homodimer have also been constructed through “click” chemistry-mediated fusion of two different alkynylated D_2_R agonist propyl aminoindane (IndAlk or IndBipAlk) utilizing PEG linkers (mitigating the lipophilicity of alkyl linkers).^47^ Compared to monomeric alkyne **6** (D_2_R K_*i*_ = 970 nM, Figure 5), compound **7**, with a long PEG_5_-linker, was found to be more potent in a radioligand displacement assay, displaying a 16 times lower K_*i*_ value (D_2_R K_*i*_ = 59 nM). IndAlk-based homobivalent ligands displayed experimental cooperativity (Hill slope) near or below 1.0 nH, indicating a lack of bivalent behavior.

**Figure 5:**
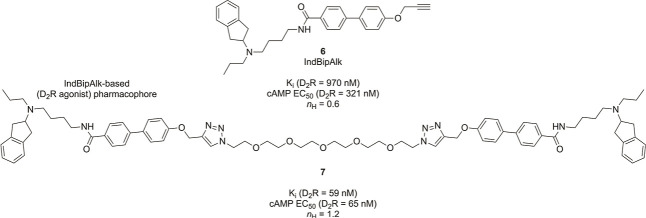
Chemical structures of IndBipAlk-based monovalent and homobivalent ligands for D_2_R.

Derived from the D_2_R/D_3_R agonist 5-OH-DPAT, a series of bivalent ligands for D_2_R/D_3_R have been developed by linking two pharmacophoric head groups via a methylene spacer of varying lengths (2, 4–7, 9–10, 12, and 14 methylene units).^48^ The optimal length for D_2_R was seen with a linker length of ten methylene units (**9**, Figure 6) (D_2_R [K_*i*_ = 2.0 nM, EC_50_ = 0.44 nM, *E*_max_ = 97%], D_3_R [K_*i*_ = 1.8 nM, EC_50_ = 0.94 nM, *E*_max_ = 74%]), whereas for D_3_R, it was with nine methylene units (**8**) (D_2_R [K_*i*_ = 2.5 nM, EC_50_ = 1.7 nM, *E*_max_ = 100%], D_3_R [K_*i*_ = 0.9 nM, EC_50_ = 0.53 nM, *E*_max_ = 92%]) (EC_50_ based on stimulation of [^35^S]­GTPgS binding to hD_2_R and hD_3_R expressed in CHO cells). The greater than one Hill numbers of **8** (1.3) and **9** (1.4) strongly indicate cooperative interaction at the two orthosteric binding sites in the homodimeric receptor.

**Figure 6:**
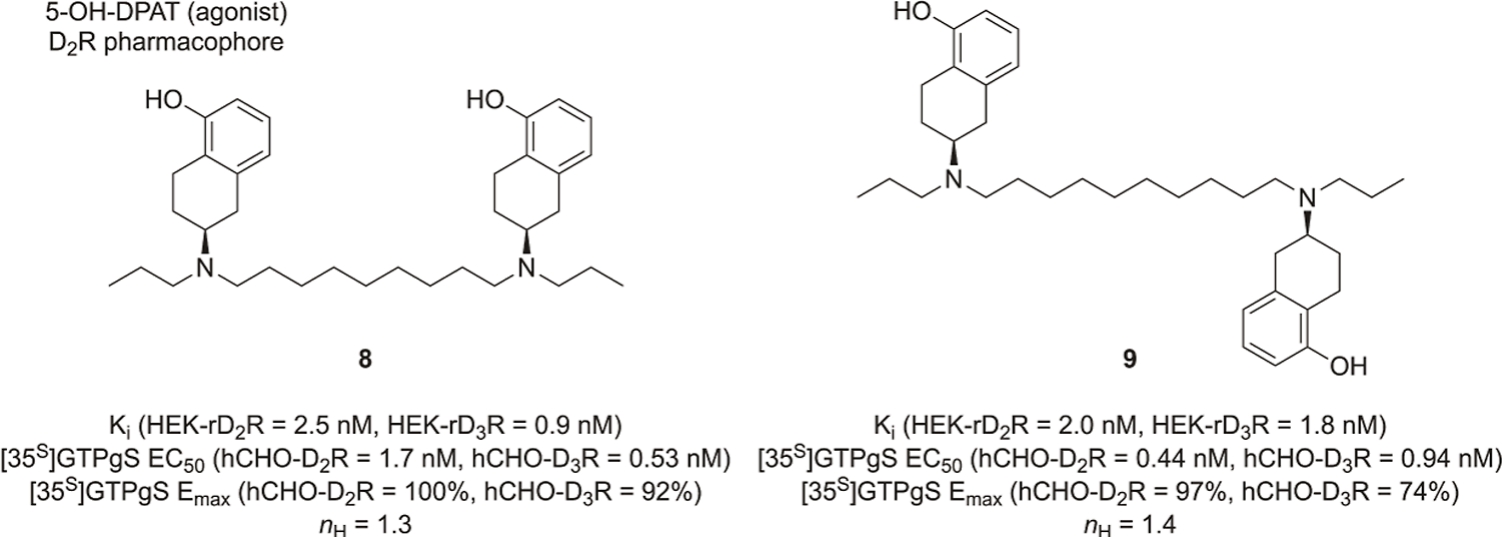
Chemical structures of 5-OH-DPAT-based homobivalent ligands for D_2_R.

The pharmacophore *N*-(*p*-aminophenethyl)­spiperone, a substructure of the D_2_R antagonist spiperone, was functionalized with a succinic acid linker to facilitate its incorporation into a bivalent system via an oligoethylene glycol (OEG) spacer.^49^ Nitrilotriacetic acid was used as a scaffold to allow for the attachment of up to three chemical entities.^50^ Compared to monovalent ligand **10** (Figure 7), bivalent ligand **11** enhances the binding affinity by 37-fold, suggesting simultaneous binding at both protomers. Utilization of synthetic peptides with the amino acid sequences of TM domains of D_2_R enabled confirmation of a bivalent binding mode ex vivo, as the administration of the disturber peptide TAT–TM6 decreased the binding of bivalent ligand **11** by 52-fold but not that of monovalent ligand **10**.

**Figure 7:**
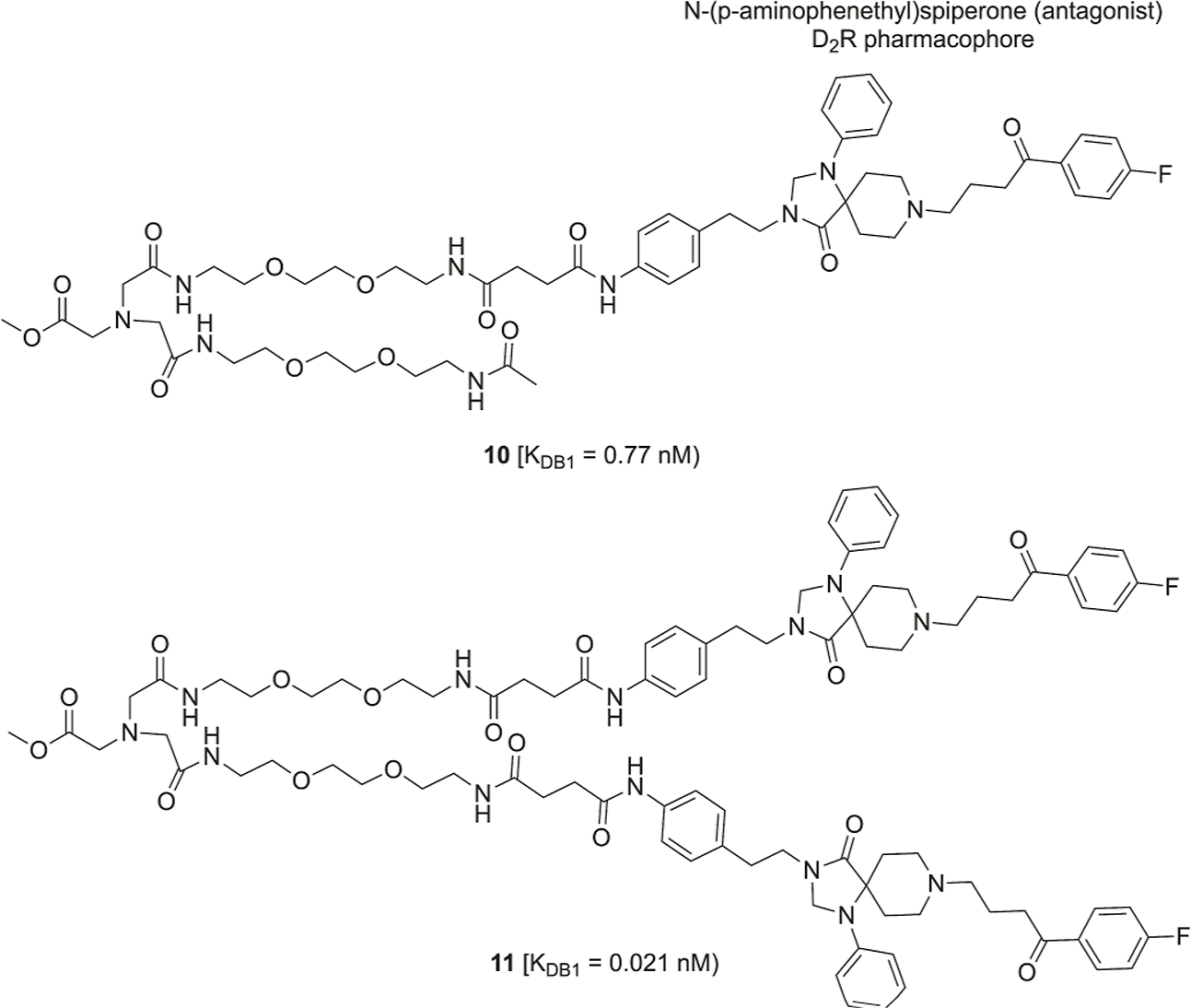
Chemical structures of spiperone-based homobivalent ligands for D_2_R.

### 2.2 DR Heterodimer-Targeting Ligands

Neurotensin receptor 1 (NTS_1_) is primarily expressed in the CNS and gastrointestinal tract and mediates multiple biological processes (including dopamine transmission and GABAergic system modulation).^51,52^ NTS_1_ is activated by the endogenous neuropeptide neurotensin (NT), pELYENKPRRPYIL; the last six residues of NT (NT8–13) are the primary epitope responsible for high-affinity receptor binding and activation.^53^ NTS_1_ and D_2_R have been shown to have physical intramembrane interaction;^54,55^ thus, bivalent ligands could be used as chemical tools to further investigate D_2_R/NTS_1_R heterodimers. High-resolution crystal structures of GPCR–ligand complexes allowed for the structure-guided design of bivalent ligands for D_2_R/NTS_1_R heterodimers.^56^ The bivalent ligands for D_2_R/NTS_1_R were constructed by combining the NTS_1_R agonist NT(8–13), the active fragment of the neuropeptide neurotensin, with three different D_2_R-specific pharmacophores [eticlopride (antagonist), 2-methoxyphenylpiperazine (antagonist), and aminoindane (agonist)] via a polyethylene glycol-based linker. Compared to cells that only express D_2_Rs, compounds **12**–**20** (Figure 8) show binding affinity in the picomolar range for cells coexpressing both GPCRs and unprecedented selectivity (up to 3 orders of magnitude). Despite the fact that bivalent ligands **18** and **19** contain pharmacophoric moieties that are functional agonists (for each GPCR), a functional switch was observed where these compounds inhibited cAMP formation in cells singly expressing D_2_Rs (behaving as an agonist) but stimulated cAMP accumulation in D_2_R/NTS_1_R-coexpressing cells. This phenomenon is not fully understood and highlights the complexity of the pharmacology of GPCR multimers. In D_2_R/NTS_1_R-coexpressing cells, the NTS_1_R protomer appears to dominate β-arrestin-2 recruitment as in the case with G protein-coupling signaling.

**Figure 8:**
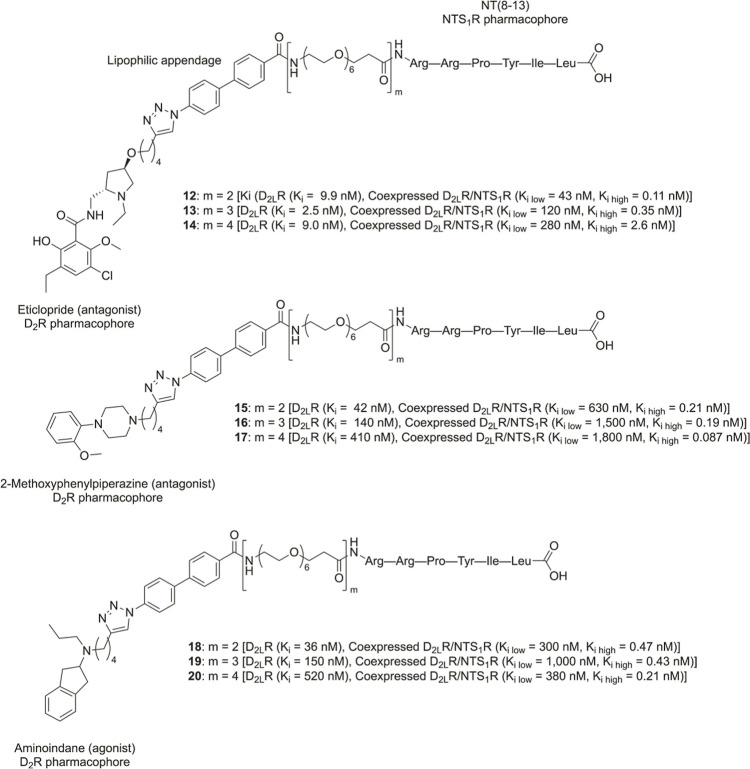
Chemical structures of bivalent ligands for the D_2_R/NTS_1_R heteromer.

Antagonistic interactions between A_2A_R and D_2_R have been observed in the corpus striatum, the primary input structure of the subcortical basal ganglia, and a key component of reward and motor systems.^57–59^ In addition, the activation of A_2A_R in the basal ganglia reduces D_2_R recognition, coupling, and signaling, resulting in increased activity of GABA neurons (via the indirect pathway), thereby reducing motor and reward functions.^60^ Thus, A_2A_R antagonists could be used as part of anti-Parkinson therapy through the enhancement of striatal D_2_R signaling. This is exemplified by the FDA approved drug, istradefylline, an A_2A_R antagonist used in combination with levodopa/carbidopa for the treatment of off-episodes in Parkinson’s disease patients.^61^

A number of biophysical experiments, including FRET and BRET analysis of living cells, have established A_2A_R–D_2_R heteromerization.^62–64^ As such, A_2A_R-antagonist/D_2_R-agonist heterobivalent ligands are valuable pharmacological tools to study A_2A_––D_2_ receptor heteromers. Composed of an adenosine A_2A_ antagonist (XCC) and a D_2_R agonist ((±)-PPHT-NH_2_) linked through a spacer of variable size (based on trifunctional amino acids in combination with PEG-polyamide unit repeats, from 26 to 118 atoms), a family of heterobivalent ligands was designed and synthesized to study the A_2A_R–D_2_R heteromer (which are implicated in Parkinson’s disease).^65^ The shorter heterobivalent ligands with linkers Lys-Lys-[PEG/polyamide]_n_-Lys-Glu (*n* = 0–3) (**21**–**24**, Figure 9) exhibited higher affinity than the corresponding monovalent controls in cells coexpressing both receptors and brain striatum tissue. This difference in affinity between monovalent and bivalent ligands was not detected in experiments from cells expressing only one receptor, indicating that the heterobivalent ligands have simultaneous interaction with both receptors of the A_2A_R–D_2_R heteromers. Expanding this work led to the development of a true heterobivalent ligand (**25**) for the A_2A_R–D_2_R heteromers that is able to favor the stabilization of the A_2A_R–D_2_R heteromer.^66^ This heterobivalent ligand is composed of succinic acid-functionalized *N*-(*p*-aminophenethyl)­spiperone (D_2_R pharmacophore) linked via an oligoethylene glycol (OEG) spacer and a nitrilotriacetic acid-based scaffold to an A_2A_R pharmacophore (a derivative of selective antagonist SCH-442,416).

**Figure 9:**
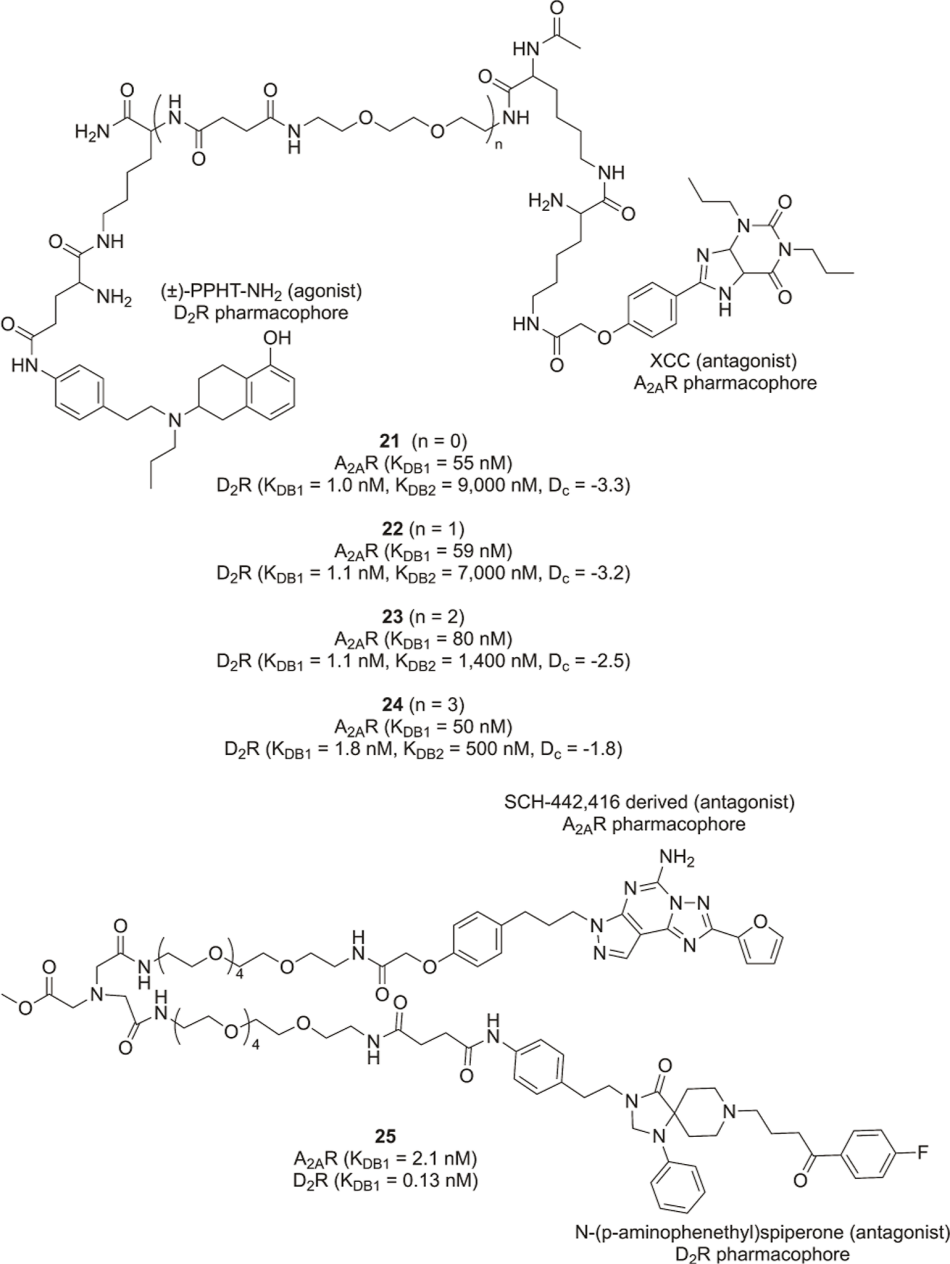
Chemical structures of bivalent ligands for the A_2A_R–D_2_R heteromer.

D_2‑l*i*ke_R and μ-opioid receptor (μOR) heterodimers are implicated as therapeutic targets for the treatment of chronic pain and addiction, as evidenced by studies demonstrating cross-regulation and intermolecular receptor–receptor interactions between μOR and D_2‑l*i*ke_R.^67–69^ Thus, μOR/D_2‑l*i*ke_R bivalent ligands can be used as pharmacological tools to investigate μOR–D_2‑l*i*ke_R heterodimers.^69^ These heterobivalent ligands were constructed by linking either a D_2‑l*i*ke_R agonist (5-hydroxy-2-(dipropylamino) tetralin, DPAT) or an antagonist (1,4-disubstituted aromatic piperazines, DAPs) to a μOR agonist (hydromorphone) or an antagonist (naltrexone) with a PEG spacer of variable length. Only compound **26** (Figure 10) displayed a biphasic competition binding curve for the D_4_R–μOR heterodimer (K_*i*_ high 1.2 nM, K_*i*_ low 207 nM), indicative of a bivalent binding mode. All the μOR/D_2‑l*i*ke_R bivalent ligands exhibited a monophasic competition binding curve to D_2_R–μOR (suggesting monovalent binding mode), potentially due to the suboptimal nature of the linkers or differences in the ligand binding kinetics of the heterodimers.

**Figure 10:**
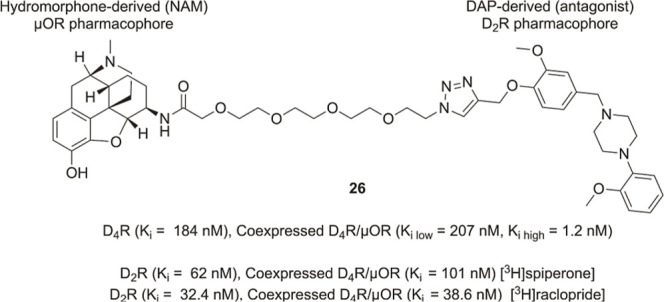
Chemical structures of a bivalent ligand for the μOR–D_2_R heteromer.

Excessive activation of metabotropic glutamate receptor 5 (mGluR5) is associated with various neurodegenerative diseases (e.g., Alzheimer’s and Parkinson’s diseases) and CNS disorders (e.g., anxiety, depression, drug addiction, and neuropathic pain).^70^ mGluR5–D_2_R–A_2A_R heteromers are potential targets for the treatment of drug addiction, locomotion, and neuropsychiatric disorders. Thus, mGluR5 NAM (NAM)–D_2_R agonist/antagonist bivalent ligands can be used as pharmacological tools to study mGluR5–D_2_R heteromers. DPAT/3-[(2-methyl-4-thiazolyl)­ethynyl]­pyridine (MTEP, mGluR5 NAM)-based bivalent ligand **27** (Figure 11) containing a 20-atom alkylamine spacer, with a protonatable nitrogen atom to mitigate lipophilicity, was found to have the best affinity for D_2_R (50 ± 6.4 nM).^70^ For cells coexpressing D_2_R and mGluR5, **27** was shown to display higher affinity to both D_2_R and mGluR5 (compared to cells monoexpressing either D_2_R or mGluR5), indicating a bivalent binding mode.

**Figure 11:**
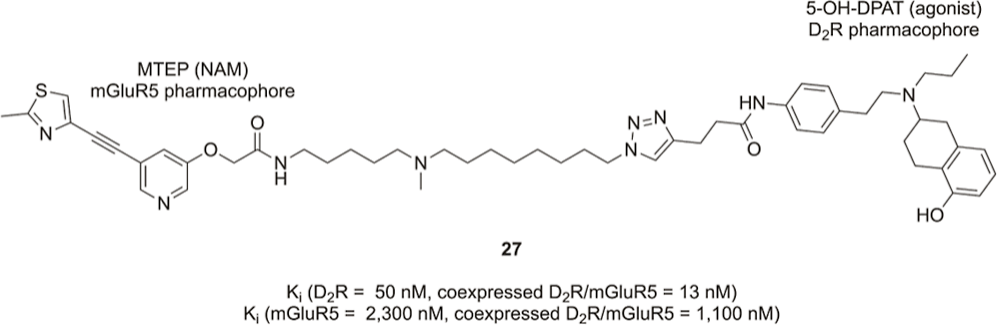
Chemical structures of the bivalent ligand for the mGluR5–D_2_R heteromer.

## 3 Fluorescent Probes

Fluorescence probes are powerful tools for using light to study cell biology, allowing for the visualization of receptors in cells and tissues and the exploration of drug–receptor interactions through biophysical techniques.^71,72^ The development of fluorescence probes has progressed significantly, but challenges remain, including the need for improved selectivity, stability (minimizing photobleaching), and permeability.

A fluorescence-based toolbox has been developed for the visualization of D_1_R and D_5_R, derived from the D_1_R antagonist SCH-23390 with a short (PEG-based) alkyl linker labeled with two different fluorescent dyes (either 5-TAMRA or DY549-P1).^73^ Pharmacological characterization revealed UR-NR435 (**28**, Figure 12), with the highest affinity [p*K*_*i*_ (D_1_R) = 8.34, p*K*_*i*_ (D_5_R) = 7.62] and selectivity toward D_2_-like receptors in radioligand binding studies. As a neutral antagonist at the D_1_R and D_5_R (via the G_s_ heterotrimer dissociation assay), UR-NR435 resists receptor internalization and degradation when working with whole cells. In kinetic binding studies using confocal microscopy, UR-NR435 exhibited rapid association and complete dissociation to D_1_R, allowing for the verification of its applicability for fluorescence microscopy. Furthermore, in agreement with radioligand binding data, molecular brightness studies of UR-NR435 revealed a single-digit nanomolar binding affinity. As such, it is evident that the fluorescent ligand UR-NR435 is an effective and versatile tool for the sophisticated characterization and investigation of native D_1_ receptors in a wide variety of experimental setups.

**Figure 12:**
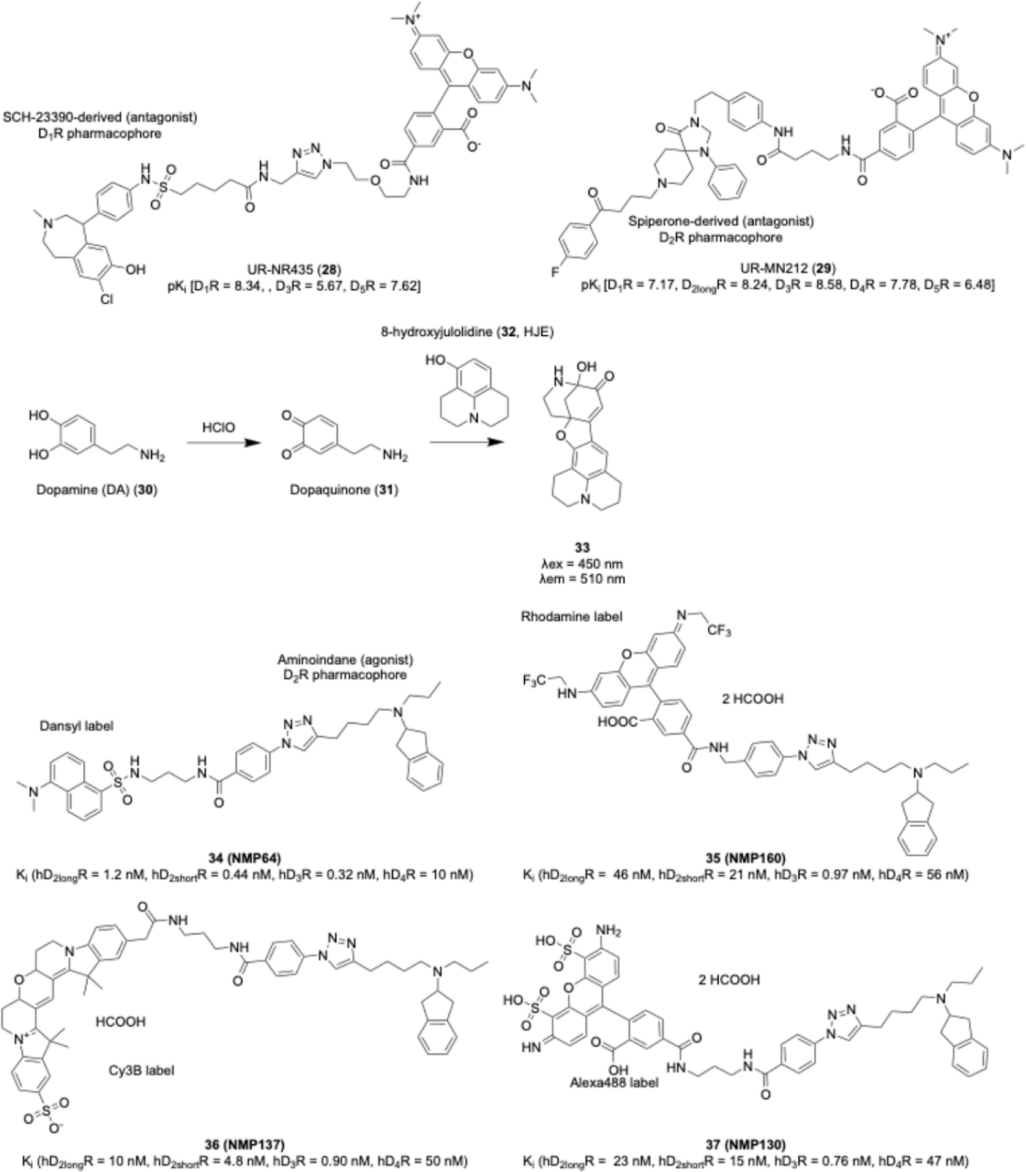
Chemical structures of dopaminergic fluorescence probes.

A fluorescence-based toolbox for imaging and ligand screening of D_2_-like receptors has also been developed, derived from the nonselective D_2_-like antagonist Spiperone with a short (γ-aminobutyric acid–based and/or PEG-based) alkyl linker labeled with two different fluorescent dyes (either 5-TAMRA or DY-D549-P1).^74^ This pharmacophore was chosen as it has high affinity for D_2_-like receptors and high selectivity compared to D_1_-like receptors (although it also has high affinity with 5-HT_1A_, 5-HT_1F_, 5-HT_2A_, α_1A_, α_1B_, and α_1D_ receptors). Furthermore, the addition of bulky structures to the aniline moiety is well tolerated allowing for an attachment point for the linker.^49,75–77^ Pharmacological characterization identified UR-MN212 (**29**, Figure 12, γ-aminobutyric acid-based linker, 5-TAMRA dye) as a high-affinity ligand for D2-like receptors [p*K*_*i*_ (D_2long_R) = 8.24, p*K*_*i*_ (D_3_R) = 8.58, p*K*_*i*_ (D_4_R) = 7.78] with reasonable selectivity toward D_1_-like receptors [p*K*_*i*_ (D_1_R) = 7.17, p*K*_*i*_ (D_5_R) = 6.48] in radioligand binding studies. In kinetic binding studies using confocal microscopy, UR-MN212 displayed rapid association to D_2long_R, allowing for the verification of its applicability for fluorescence microscopy. Molecular brightness studies of UR-MN212 revealed single-digit nanomolar binding affinity, in good agreement with radioligand binding data.

Under oxidizing conditions with hypochlorous acid (HClO), a common reactive oxygen species in biological systems, DA (**30**, Figure 12) can be oxidized to dopaquinone (**31**) and subsequently coupled to 8-hydroxyjulolidine (**32**, HJE) to yield a highly emissive and bathochromically shifted fluorescent derivative HJE-DA (**33**).^78^ Compound **33** was used as a probe to detect and image DA in inflammation and depression cell models, marking the first method to use an organic fluorescent probe to detect DA in living cells.

The fluorescent DR ligand NAPS (*N*-azidophenethylspiperone)–DY-647 (chemical structure undisclosed) was used in FRET-based binding assays, enabling the discovery of a single, divergent glycine in extracellular loop 1 as the critical determinant for the pharmacological specificity of D_2_R and D_3_R.^79^ Likewise, a series of fluorescent ligands (**34**–**37**, Figure 12) for D_2_R and D_3_R was synthesized to enable NanoBRET binding assays and visualization of DRs on the surface of living cells by total internal reflection microscopy.^80^

Fluorescent dopamine nanosensors enable localized mapping of dopamine release and reuptake with micrometer-level spatial resolution.^81^ For instance, nIRCat is a near-IR sensor that allows for the detection of specific neurotransmitter release (e.g., dopamine).^82^ nIRCat is a functionalized single-walled carbon nanotube that undergoes a 10-fold increase in fluorescence in the presence of dopamine, enabling dopamine imaging in brain slices from wild-caught mice. This technology is highly specific; a change in fluorescence is not observed for acetylcholine, gamma-aminobutyric acid (GABA), or glutamate, whereas fluorescence is observed for norepinephrine, but with a lower affinity and maximal response (thus nonproblematic). Similarly, AndromeDA is a single-walled carbon nanotube near-infrared fluorescent dopamine sensor that can simultaneously visualize dopamine release for up to 100 dopaminergic varicosities (in conjunction with a machine learning-based analysis tool).^83^

As an alternative to the nanoparticle approach for imaging dopamine, a genetically targeted protein sensor can be used. Cell-based neurotransmitter fluorescent-engineered reporters (CNiFERs) were originally developed to detect acetylcholine, in which the G_q_ protein-coupled receptor was expressed with a FRET-based Ca^2+^ sensor TN-XXL.^84^ For dopamine sensing, D_2_R is transfected as a modified G_q/*i*5_ protein chimera that recruits the G_q_ signaling pathway (despite D_2_R being a G_*i*/o_-coupled receptor).^85^ Dopamine binds to the modified D2R receptors, which activate G_q/*i*5_ and, in turn, the PLC/IP_3_ cascade that results in the release of stored Ca^2+^.^85^ The release of Ca^2+^ results in a conformational shift in TN-XXL and increases the FRET ratio (Δ*R*/R), which can be monitored using fiber photometry in vivo.

cpGFP-calmodulin is composed of a myosin light chain RS20 peptide on the N-terminus and a circularly permutated green fluorescent protein fused to the calcium-binding motif on the C-terminus.^84^ Calcium induces a conformational change that forces the GFP into a fluorescent conformation, thus enabling investigation into neuronal activity by measuring fluorescence in vivo by an optical fiber that is implanted in the brain. By expressing GCaMP in spiny projection neurons, dopamine release can be inferred through the change in intracellular calcium.^86^ Several more sophisticated constructs have been developed as genetically encoded fluorescent dopamine sensors, such as dLight and GRAB_DA_ sensors. The dLight sensor is composed of a GCaMP6 sensor inserted into the third intracellular loop of a DR, such that GFP fluorescence increases when dopamine is bound.^87–90^ Similarly, GRAB_DA_ consists of cpEGFP inserted into the third intracellular loop of the D2R.^91^

## 4 Photopharmacological Probes

Photopharmacology allows for precise spatiotemporal control, with the ability to toggle protein function off and on through modulation of a synthetic photoswitch (often photoisomerizable azobenzene) altering a ligand’s affinity and/or efficacy for its endogenous biological target.^92^ Genetic approaches (e.g., the overexpression, knockout, or knockdown of individual proteins) can also control individual DRs in specific cell types; however, understanding of the temporal aspects of DR activation is hindered as receptor function is impacted over long time scales (potentially imposing confounding compensatory effects on neuronal physiology).^93^ Light-gated DRs (LiDARs) have been engineered to be spatiotemporally modulated by a tetherable azobenzene conjugated to the synthetic D_2_R agonist 2-(N-phenethyl-N-propyl)­amino-5-hydroxytetralin (PPHT) in a receptor-specific and cell-type-specific manner.^94^ This photoswitchable tethered ligand maleimide–azobenzene–PPHT (*trans*-MAP (**38**) and *cis*-MAP (**39**); Figure 13) was covalently conjugated to an engineered cysteine through a maleimide–thiol reaction, allowing for selective remote control of DRs using light. It should be noted that due to the potential for off-target labeling and instability toward hydrolysis, maleimide–thiol chemistry has limited in vivo applications (although it does work well in cell culture). Untethered azobenzene-containing photochromic ligands (PCLs) can photoswitch Family A GPCRs, including DRs (*trans*-AP, **40**, *cis*-AP, **41**), but these ligands bear the caveat of off-target effects and slower kinetics compared to their tethered counterparts.^94–96^

**Figure 13:**
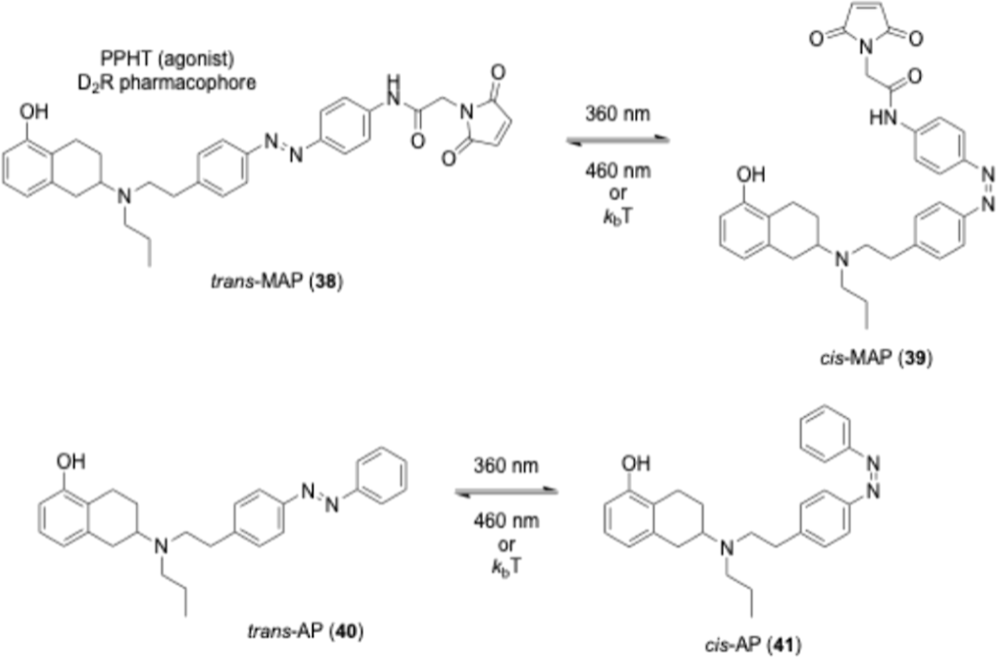
Chemical structures of maleimide–azobenzene–PPHT (MAP).

To improve upon the limitation of maleimide–thiol chemistry, membrane-anchored photoswitchable orthogonal remotely tethered agonist and antagonist were developed for the DR, benzylguanine-azobenzene-PPHT [*trans*-p-D1_ago_ (**42**) and *cis*-p-D1_ago_ (**43**); Figure 14][Bibr ref97] and benzylguanine-azobenzene-SKF83566 [*trans*-p-D1_block12_ (**44**), *trans*-p-D1_block24_ (**45**), *cis*-p-D1_block12_ (*n* = 12, **46**], and *cis*-p-D1_block24_ (*n* = 24, **47**).^98^ The benzylguanine (BG) moiety selectively and covalently reacts with SNAP-tag, which is anchored to the plasma membrane via a single-pass transmembrane segment (allowing for precise targeting of dorsal striatal medium spiny neurons). This technology (photoswitchable orthogonally tethered ligand -PORTL), was further extended to D_2_-like receptors using a HaloTag membrane anchor and D_2_-like receptors’ selective pharmacophores (**48**–**53**, Figure 15).^99^ As both SNAP-tag and HaloTag are orthogonal to each other, multiplexed experiments could be conducted wherein SNAP-M for D_1_-_l*i*ke_R and Halo-M for D_2‑l*i*ke_R can be controlled independently.^98^

**Figure 14:**
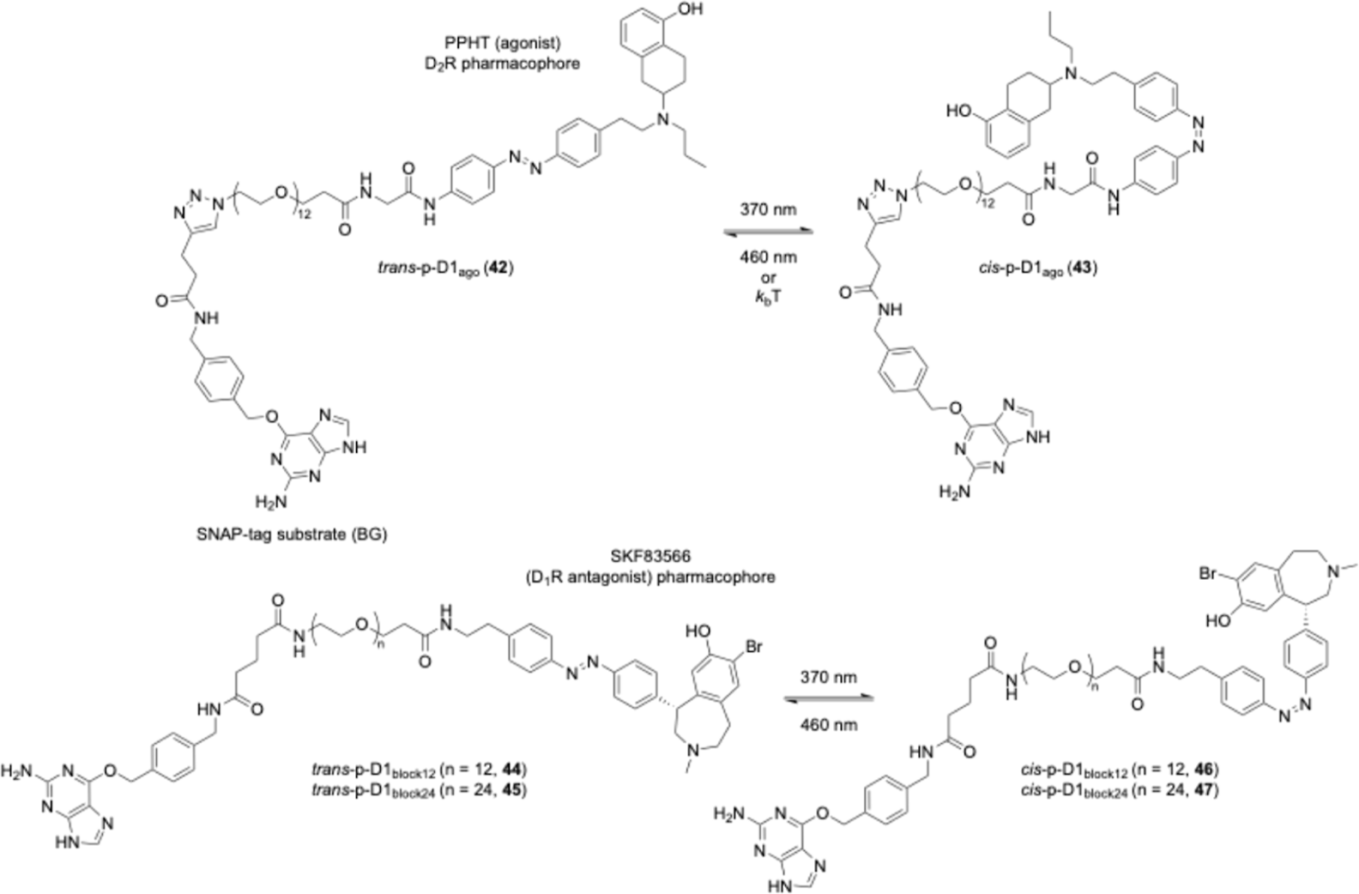
Chemical structures of benzylguanine–azobenzene–PPHT and benzylguanine–azobenzene–SKF83566.

**Figure 15:**
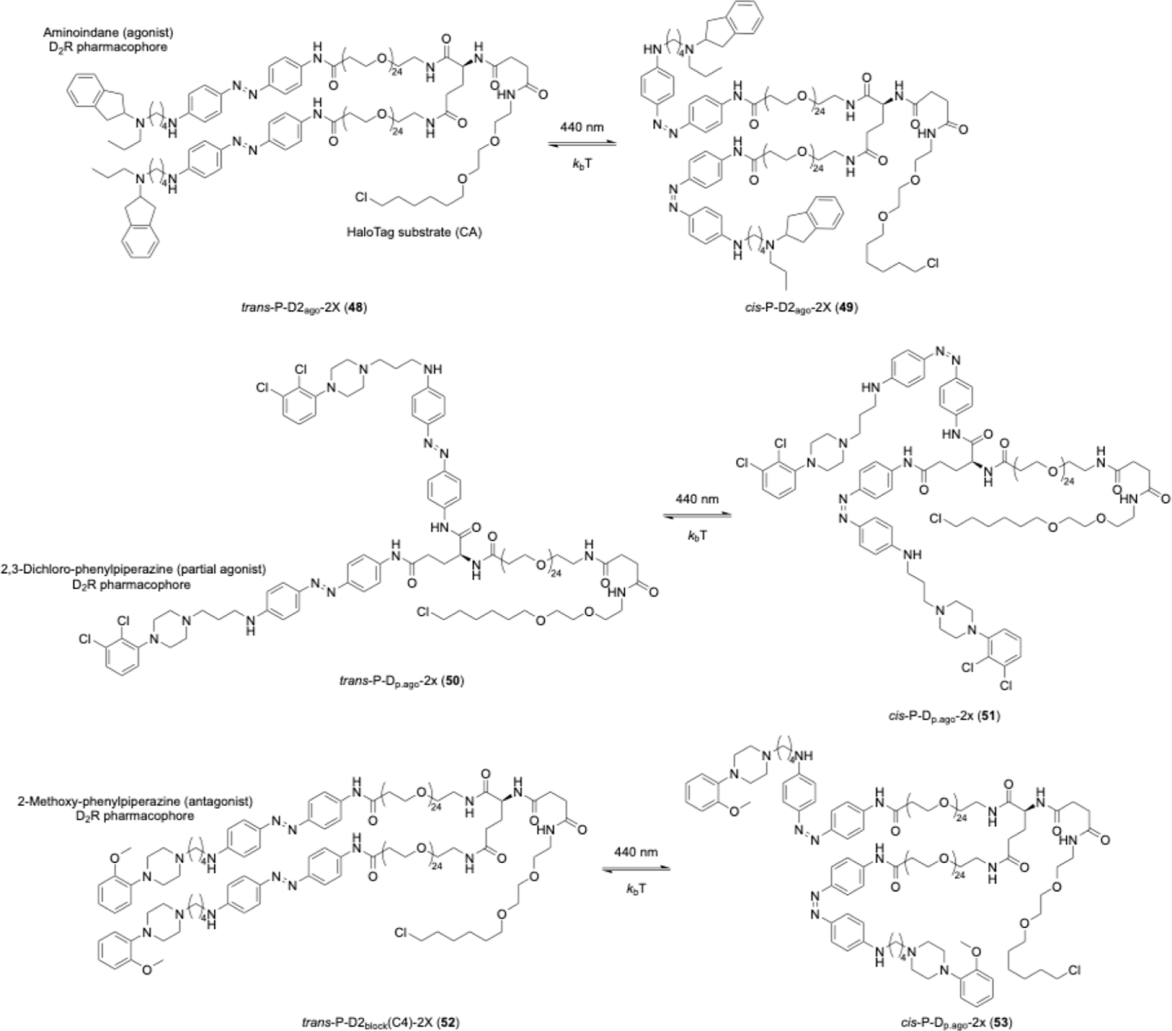
Chemical structures of HaloTag membrane-anchored photoswitchable dopamine ligands.

Compared to photoisomerizable compounds, which reversibly change activity with light, photoactivatable (caged) compounds are irreversibly activated by photolysis through the release of a masking group. Photoactivatable dopamine (CyHQ-O–DA, **54** and **55**; Figure 16) and the selective D_2_R antagonist sulpiride (CyHQ-sulpiride, **56**) enabled the detailed study of the function of dopaminergic neurons and circuits.^100^ This is because the precise simulation of presynaptic dopamine release and tight temporal control over the release of DR antagonists are essential for investigating dopamine-dependent signaling as well as for the kinetic analysis of DR activation and inactivation. Caged versions of dopamine and sulpiride were constructed using 8-cyano-7-hydroxyquinolinyl (CyHQ) as a photoremovable protecting group, which can be deprotected with either 1-photon excitation (365 or 405 nm light) or 2-photon excitation (740 nm light) processes.

**Figure 16:**
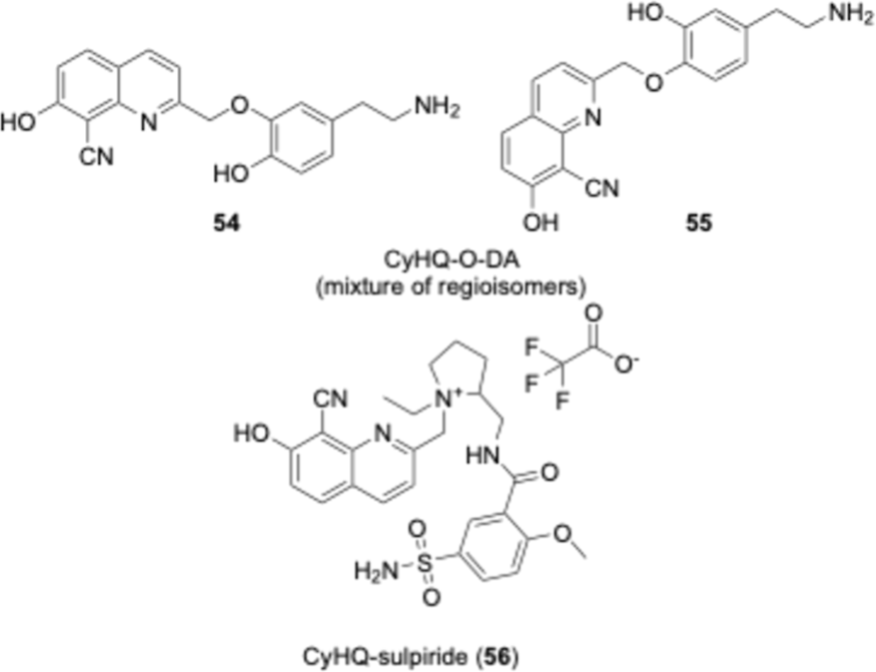
Chemical structures of dopaminergic photoactivatable probes.

Photoaffinity probes were developed to covalently bind to D_2_R and provide a handle for affinity purification or detection, potentially allowing insight into binding interactions (for both D_2_R and other off-target proteins).^101^ This could be particularly insightful as limited structural data regarding D_2_R exists, requiring extensive mutation (to the point of altering the receptor’s ligand binding) to enable temperature stability and easier isolation.^102,103^ The probes were designed by incorporating ropinirole and pramipexole pharmacophores with benzophenone, a photo-cross-linking group, and an alkyne (**57** and **58**, Figure 17). The alkyne can undergo copper­(I)-catalyzed alkyne–azide cycloaddition (CuAAC) “click” chemistry with either an azide fluorophore, enabling microscopy and flow cytometry analysis, or an azide enrichment tag, enabling proteomics and mass spectrometry analysis.

**Figure 17:**
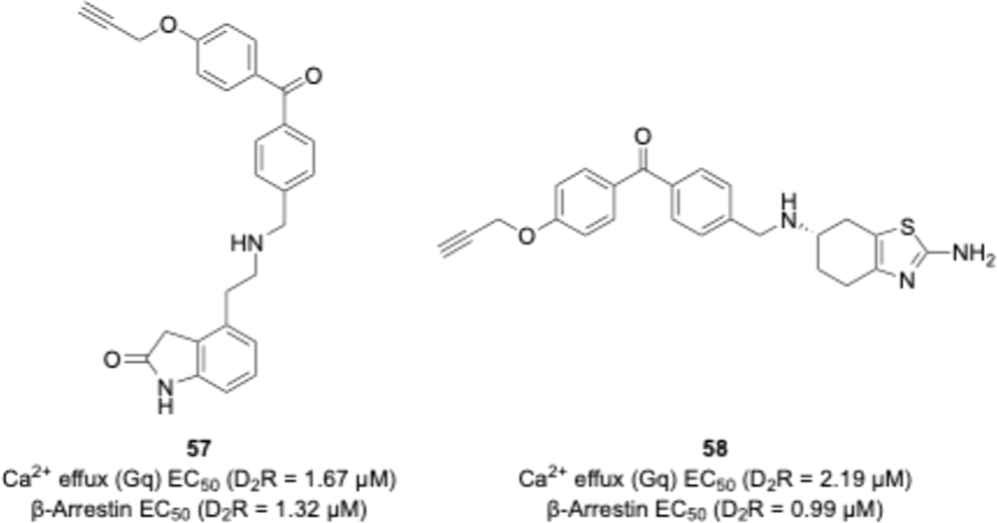
Chemical structures of dopaminergic photoaffinity probes.

## 5 Conclusion and Perspectives

As potential therapeutic agents in the treatment of numerous neurological diseases and disorders, dopamine ligands can revolutionize the field of medicine, as they already have proven clinical utility with pharmaceuticals like l-DOPA and antipsychotics (e.g., pimozide, haloperidol, chlorpromazine, olanzapine, and risperidone). Despite their prevalent clinical use, conventional dopaminergic ligands are plagued with incapacitating side effects due to insufficient drug-like properties and selectivity (between dopamine subtypes and related biogenic amine receptors). Likewise, insignificant knowledge of the pharmacodynamic properties of DRs limits the development of therapeutics with enhanced synergic interactions (e.g., based on protein/protein interactions with the receptor oligomer) or selective biased signaling activation. The ever-expanding chemical toolkit (e.g., bivalent, fluorescent, photoactivatable, and photoaffinity probes; Table 1) is only beginning to reveal critical information on the unique pharmacology of DRs and will continue to grow, aided in part by new developments in mass spectrometry-based proteomics and computational methods. As demonstrated by DR heterodimer-targeting ligands, DRs are not isolated systems; thus, the approaches addressed here could be extended to the dopamine transporter (DAT) and other dopamine-regulatory proteins, as well as to other dopamine-interacting neuromodulatory systems, such as serotonergic and noradrenergic pathways, to probe receptor crosstalk.

**Table 1:** Summary of the Chemical Tools for DRs

receptor(s)	tool paradigm	compound number
D_2_R homodimer	bivalent ligands	**1**, **2**, **7**, **8**, **9**, **11**
D_2_R/D_3_R	bivalent ligands	**8**, **9**
D_2_R/NTS_1_R heterodimer	bivalent ligands	**12**–**20**
A_2_AR–D_2_R heterodimer	bivalent ligands	**21**–**25**
μOR–D_2_R heterodimer	bivalent ligands	**26**
mGluR5–D_2_R heterodimer	bivalent ligands	**27**
D_1_R, D_5_R	fluorescent probes	**28** (UR-NR435)
D_2_-like receptors (D_2_R, D_3_R, D_4_R)	fluorescent probes	**29** (UR-MN212)
Not Applicable	fluorescent probes	**33** (HJE-DA)
D_2_R/D_3_R	fluorescent probes	**34**–**37**
D_1_R, D_2_R	photopharmacological probes (LiDARs/PCLs/PORTL)	**38**–**47**
DR	photoactivatable (caged) probes	**54**, **55**
D_2_R	photoactivatable (caged) probes	**56**
D_2_R	photoaffinity probes	**57**, **58**
